# Comparison of the efficacy of the Epley maneuver and repeated Dix–Hallpike tests for eliminating positional nystagmus: A multicenter randomized study

**DOI:** 10.3389/fneur.2023.1095041

**Published:** 2023-02-27

**Authors:** Takao Imai, Atsuhiko Uno, Akiyuki Yamato, Yasumitsu Takimoto, Go Sato, Kazunori Matsuda, Noriaki Takeda, Suetaka Nishiike, Kayoko Kawashima, Tomoko Iga, Yuya Ueno, Yumi Ohta, Takashi Sato, Takefumi Kamakura, Kayoko Shingai-Higashi, Shinji Mikami, Naomiki Kimura, Takashi Nakajima, Akihisa Tanaka, Hidenori Inohara

**Affiliations:** ^1^Department of Otorhinolaryngology – Head and Neck Surgery, Osaka University Graduate School of Medicine, Osaka, Japan; ^2^Department of Otorhinolaryngology – Head and Neck Surgery, Bell Land General Hospital, Osaka, Japan; ^3^Department of Otorhinolaryngology – Head and Neck Surgery, Osaka General Medical Center, Osaka, Japan; ^4^Department of Otolaryngology, Suita Municipal Hospital, Osaka, Japan; ^5^Takimoto Ear, Nose & Throat Clinic, Osaka, Japan; ^6^Department of Otorhinolaryngology – Head and Neck Surgery, Tokushima University Graduate School of Biomedical Sciences, Tokushima, Japan; ^7^Department of Otolaryngology, Tokushima Prefectural Central Hospital, Tokushima, Japan; ^8^Department of Otorhinolaryngology – Head and Neck Surgery, Osaka Rosai Hospital, Osaka, Japan; ^9^Department of Otolaryngology, Osaka Habikino Medical Center, Osaka, Japan

**Keywords:** BPPV fatigue, positional nystagmus, posterior canal, canalolithiasis, cupulolithiasis

## Abstract

**Background and objectives:**

Patients with benign paroxysmal positional vertigo of the posterior canal (pc-BPPV) exhibit BPPV fatigue, where the positional nystagmus diminishes with the repeated performance of the Dix–Hallpike test (DHt). BPPV fatigue is thought to be caused by the disintegration of lumps of otoconial debris into smaller parts and can eliminate positional nystagmus within a few minutes [similar to the immediate effect of the Epley maneuver (EM)]. In this study, we aimed to show the non-inferiority of the repeated DHt to the EM for eliminating positional nystagmus after 1 week.

**Methods:**

This multicenter, randomized controlled clinical trial was designed based on the CONSORT 2010 guidelines. Patients who had pc-BPPV were recruited and randomly allocated to Group A or Group B. Patients in Group A were treated using the EM, and patients in Group B were treated using repeated DHt. For both groups, head movements were repeated until the positional nystagmus had been eliminated (a maximum of three repetitions). After 1 week, the patients were examined to determine whether the positional nystagmus was still present. The groups were compared in terms of the percentage of patients whose positional nystagmus had been eliminated, with the non-inferiority margin set at 15%.

**Results:**

Data for a total of 180 patients were analyzed (90 patients per group). Positional nystagmus had been eliminated in 50.0% of the patients in Group A compared with 47.8% in Group B. The upper limit of the 95% confidence interval for the difference was 14.5%, which was lower than the non-inferiority margin.

**Discussion:**

This study showed the non-inferiority of repeated DHt to the EM for eliminating positional nystagmus after 1 week in patients with pc-BPPV and that even the disintegration of otoconial debris alone has a therapeutic effect for pc-BPPV. Disintegrated otoconial debris disappears from the posterior canal because it can be dissolved in the endolymph or returned to the vestibule *via* activities of daily living.

**Classification of evidence:**

This study provides Class II evidence of the non-inferiority of repeated DHt to the EM for eliminating positional nystagmus after 1 week.

**Registration number:**

UMIN000016421.

## Introduction

Benign paroxysmal positional vertigo (BPPV) is the most common cause of vertigo and is characterized by positional nystagmus (1). BPPV can be treated using the canalith repositioning procedure (CRP) (2), which is more effective than a sham maneuver (3), medication alone (4), or no treatment (4). The Epley maneuver (EM) is a type of CRP effective for treating benign paroxysmal positional vertigo of the posterior canal (pc-BPPV) (5). However, the efficacy of the EM is comparable to that of other CRPs, such as the Semont and Gans maneuvers (6). The treatment of BPPV using the EM, Semont maneuver, or Gans maneuver has immediate effects. For instance, following the EM, the characteristic positional nystagmus can no longer be observed, as assessed using the Dix–Hallpike (DH) test (1, 7). By contrast, sham maneuvers, medication alone, and no treatment do not show such effects, thus demonstrating the superiority of CRP maneuvers for treating BPPV.

The repeated performance of the DH test for assessing positional nystagmus in patients with pc-BPPV gives rise to BPPV fatigue, where the positional nystagmus and dizziness/vertigo decrease with each repetition (8). BPPV fatigue is thought to be caused by the disintegration of lumps of otoconial debris into smaller parts (9, 10). Otoconial debris must be of sufficient size to stimulate the semicircular canal during movement in the canal; small otoconial debris cannot stimulate the canal and thus does not give rise to positional nystagmus and dizziness/vertigo. This is the putative pathophysiology of BPPV fatigue. The repeated performance of the DH test can eliminate positional nystagmus, similar to the effect seen immediately after the EM. Therefore, the repeated performance of the DH test may have the same therapeutic effect as the Epley, Semont, and Gans maneuvers.

In this study, we hypothesized that repeated application of the DH test and EM will have the same efficacy in terms of eliminating positional nystagmus. We tested this by comparing the cure rates of patients with pc-BPPV after repeated application of the DH test or EM in a multicenter randomized trial. In this investigation, “cure” was defined as the elimination of positional nystagmus after 1 week. We aimed to demonstrate that repeating the DH test is equally effective as the EM for treating pc-BPPV. This would be beneficial for both clinicians and patients with pc-BPPV because DH test repetitions would be quicker and less painful than the EM because it involves fewer head movement steps.

## Materials and methods

### Standard protocol approval, registration, and patient consent

The study was conducted in accordance with the Declaration of Helsinki. Approval for the study was obtained from the ethics committee of Osaka University Hospital (No. 14128), and the study was registered in the University Hospital Medical Information Network (UMIN000016421). The study protocol is available online (11). Written informed consent was obtained from all study participants.

### Trial design

This study was a multicenter, non-blinded, randomized controlled clinical trial designed based on the 2010 Consolidated Standards of Reporting Trials guidelines (CONSORT 2010). The study was initially designed to run for 5 years, but this was extended to 8 years because of the lack of patients. No other changes were made to the study methods or design.

### Participants

The study was carried out in the otorhinolaryngology departments of several hospitals: Osaka University Hospital (Osaka, Japan), Tokushima University Hospital (Tokushima, Japan), Osaka Rosai Hospital (Osaka, Japan), Otemae Hospital (Osaka, Japan), and Suita Municipal Hospital (Osaka, Japan). We included patients diagnosed with pc-BPPV (12) based on positional nystagmus in a DH test. The pathophysiology of pc-BPPV involves canalolithiasis (13), but in certain patients, there may be cupulolithiasis (14). To limit the study to patients with canalolithiasis, patients were only included if they had positional nystagmus that lasted <1 min (14, 15). This time limit was restricted to the first phase of positional nystagmus because the direction of the nystagmus can reverse within 1 min, thereby creating a second phase and lengthening the total duration of the positional nystagmus to >1 min (16). Patients with this second phase were included if the duration of the first phase was <1 min.

Patients were excluded if they did not consent to participate or had a confirmed or possible pregnancy, severe heart disease, medical history, or the possibility of cervical/lumbar disc disease.

The patients were randomly assigned to Group A or Group B. Block randomization was carried out by our data management center. A block size of six was chosen, but this was only known to the data management center; the team members carrying out the clinical intervention were unaware of the block size to ensure that the group allocations remained unknown.

### Interventions

**Figure 1:**
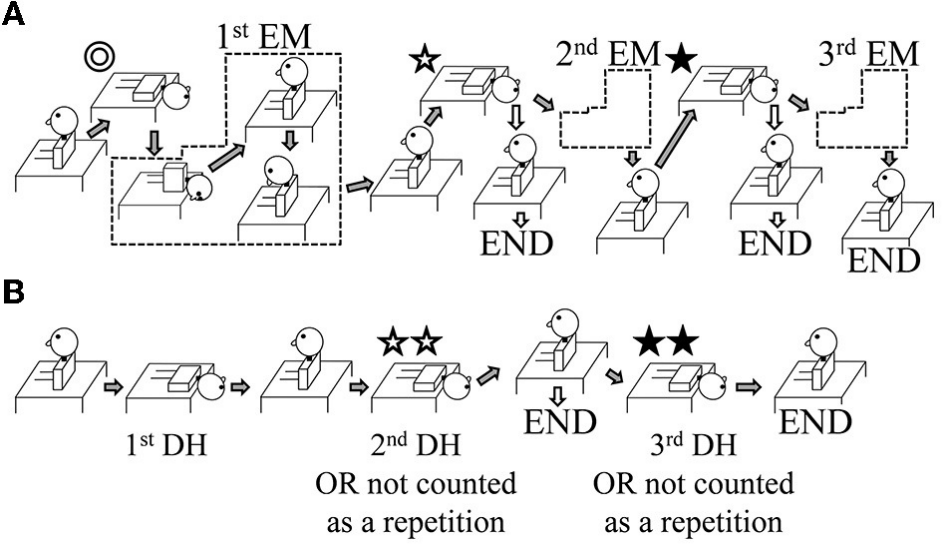
Illustration of the study interventions. The interventions shown are for patients with right-sided benign paroxysmal positional vertigo (BPPV). In the case of left-sided BPPV, lateralized movements are performed on the opposite side. **(A)** Group A: The Epley maneuver (EM) was repeated a maximum of three times. After the first and second EM repetitions, the positional nystagmus was assessed using a Dix–Hallpike (DH) test (shown by ✩ and ⋆). If positional nystagmus was absent on the affected side, the intervention ended (shown by the white arrows pointing to the word END). If positional nystagmus was still present, a further EM repetition was performed (shown by the gray arrows). **(B)** Group B: The repeated DH test was performed with up to three repetitions. If the positional nystagmus on the affected side was found to be absent at the second DH test (shown by ✩✩), the intervention ended (shown by the white arrows pointing to the word END); in this case, the number of repetitions was recorded as one, i.e., the movement shown by ✩✩ was not counted. If positional nystagmus was still present, a further DH test was performed (shown by the gray arrows). For the third DH test (shown by ⋆⋆), if positional nystagmus was absent, the number of repetitions was recorded as two, i.e., the movement shown by ⋆⋆ was not counted; if positional nystagmus was present, the number of repetitions was recorded as three.

The authors who carried out the interventions were certified to treat patients with dizziness/vertigo (certified by the Japan Society for Equilibrium Research). Participants in Group A were treated using the EM on the affected side (13, 17) (Figure 1A). Participants in Group B were treated using repetitions of the DH test (Figure 1B). During the interventions, the patients wore Frenzel glasses with an infrared charge-coupled device (CCD) camera (*IEM-2*, Nagashima Medical Instruments Co. Ltd, Tokyo, Japan) so that the positional nystagmus could be observed.

Group A: For the EM, the patient's head was moved from one position to the next when there was no longer any positional nystagmus. However, if the positional nystagmus lasted >1 min, the patient's head was moved to the next position. Similarly, for the first head position (Figure 1A ⊚, ✩, ⋆), if the direction of the positional nystagmus reversed (the second phase of nystagmus), the patient's head was moved to the next position. If positional nystagmus was not observed in a given head position, the patient's head was moved after ~30 s. Following the EM, the DH test was performed on the affected side after ~1 min (Figure 1A ✩, ⋆). If positional nystagmus was not observed or lasted <2 s, the EM treatment ended (END; see white arrows in Figure 1A); if there was positional nystagmus that lasted >2 s, the EM was repeated for a maximum of three times (second EM and third EM; see gray arrows in Figure 1A).

Group B: For the first DH test, when positional nystagmus was no longer observed, and at the start of the second phase of nystagmus, patients were returned to the sitting position. After about 30 s, a second DH test was performed (Figure 1B ✩✩). If positional nystagmus was not observed or lasted <2 s, the DH test treatment ended (END; see white arrows in Figure 1B); in this case, one repetition of the DH test was considered to have been performed because the second DH test served to confirm the elimination of positional nystagmus and would not have contributed to BPPV fatigue. If positional nystagmus was observed for >2 s during the second DH test, patients were returned to the sitting position, and a third DH test was performed after about 30 s (Figure 1B ⋆⋆). If positional nystagmus was not observed or lasted <2 s in this third test, the number of repetitions in the DH test treatment was taken as two because the test served to confirm the elimination of positional nystagmus and would not have contributed to BPPV fatigue; however, if positional nystagmus was observed for >2 s, the number of repetitions in the DH test treatment was taken as three.

Exactly 1 week after the intervention, the patients returned to the hospital. The DH test was carried out, followed by the supine roll test (1). For these tests, patients wore the Frenzel glasses with an infrared CCD camera so that the positional nystagmus could be observed. A patient was considered to be cured if positional nystagmus was not observed in the DH test on the affected side. If positional nystagmus characteristic of lateral canal BPPV (lc-BPPV) (15), but not of pc-BPPV, was observed, then the participants were still considered to be cured even though the cure was restricted to pc-BPPV; this situation may occur when otoconial debris in the posterior semicircular canal moves to the lateral canal (the “canal switch”) (18). When positional nystagmus was observed in the DH test on the contralateral side but not on the affected side, the patient was also considered to be cured even though the cure was restricted to the side that was originally affected. For convenience, we also refer to this situation as a canal switch. However, patients were not considered to be cured when positional nystagmus was observed in the DH test on the affected side, which is characteristic of pc-BPPV even when the nystagmus was only mild. To summarize, a curative effect was only considered when the positional nystagmus characteristic of pc-BPPV was eliminated on the affected side.

Following this session, no further interventions were performed, and the patients received normal medical care.

### Outcomes

#### Primary outcome

The primary outcome was the pc-BPPV cure rate.

#### Secondary outcomes

One secondary outcome was the number of treatment repetitions using the EM (Group A) or DH test (Group B). Another secondary outcome was the distribution of BPPV subtypes in Groups A and B after 1 week. The ipsilateral pc-BPPV subtype indicated that a patient was not cured; other subtypes (contralateral pc-BPPV, lc-BPPV with geotropic positional nystagmus, and lc-BPPV with apogeotropic positional nystagmus) were taken to indicate that a patient's pc-BPPV was cured.

### Blinding

Patients were not told whether they were allocated to Group A or Group B. However, when informed consent was obtained, the head movements for the EM and DH tests were explained, and thus, some patients may have been aware of their group assignment. After the final session assessing positional nystagmus, 1 week after the intervention, patients were told whether they had been allocated to Group A or B. The physicians who assessed the positional nystagmus at the final session also enrolled the patients; it is, therefore, possible that they remembered the patients' group allocations. However, this would appear to be unlikely as this information was not recorded in the medical records and a considerable number of patients are seen within a week.

### Sample size

The sample size was determined based on the assumption that (the cure rate for the EM) - (the cure rate for the repeated DH test) would be <15% if they have equivalent efficacy. In other words, the non-inferiority margin was set at 15%. This margin was chosen based on our previous study, which showed a cure rate for the EM of ~80% (19) compared to 50% when there was no CRP. The difference between these cure rates was 30% (50–80), and the non-inferiority margin was set to half of this value, i.e., 15%.

The sample size was determined based on the following conditions:

Primary outcome: cure rate (the number of patients whose positional nystagmus due to pc-BPPV was cured on the affected side after 1 week)/(the number of patients in each group).

Analysis method: a non-inferiority trial using cure rates

Difference between the groups: 80% – 80% = 0%

Non-inferiority margin: 15%

Type I error, α: 5% (one-tailed test)

Power, 1 – β: 80%


$$\begin{array}{l} n=\frac{{\left\{1.645\sqrt{0.875\left(1-0.875\right)+\left(0.875-0.15\right)\left(1-0.875+0.15\right)}+0.842\sqrt{2\cdot 0.8\left(1-0.8\right)}\right\}}^{2}}{0.15^{2}}=85.9 \end{array}$$

Finally, we rounded this value up to give a sample size of 90 patients per group.

### Statistical methods

The statistical analyses were performed using BellCurve for Excel (Social Survey Research Information Co., Ltd., Tokyo, Japan) and EZR software (20). One-tailed Z-tests were used to analyze group differences in the cure rate (primary outcome), sex ratio, and laterality ratio of the BPPV; a one-tailed *t*-test was used to analyze differences in patient age. The secondary outcomes were analyzed using Fisher's exact test. Statistical significance was defined as a *p-*value of < 0.05.

### Data availability

The data supporting the findings of this study are available from the corresponding author upon reasonable request.

The study protocol and statistical analysis plan are available in eSAP1.

## Results

**Figure 2:**
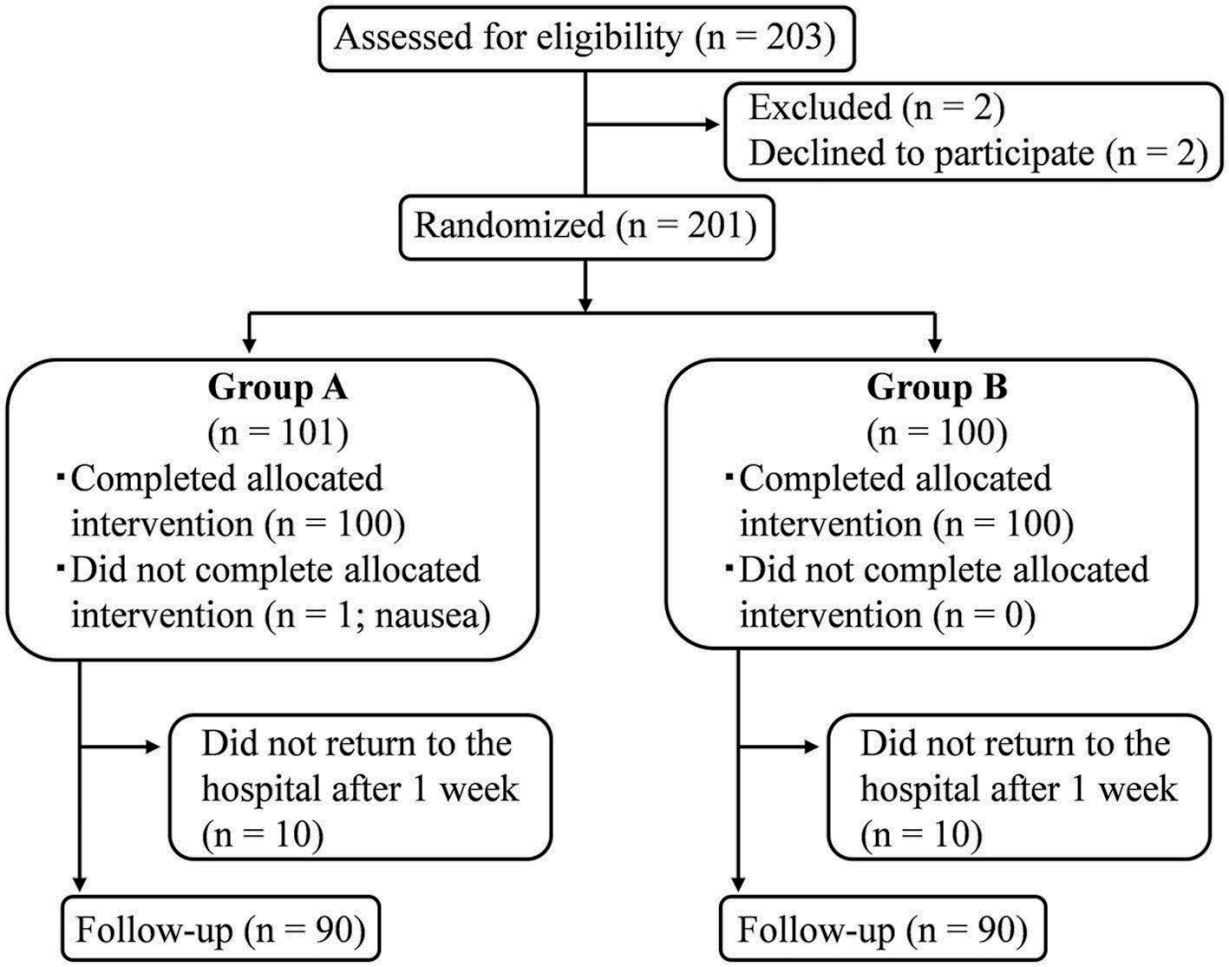
Flow diagram of patient inclusion, allocation, and follow-up. A total of 180 patients completed the study, including the final assessment that determined whether positional nystagmus was still present.

Patients with pc-BPPV were recruited in the study between 8 January 2015 and 9 December 2021. Data acquisition was completed for a total of 180 patients (90 per group) by 16 December 2021. A flowchart detailing patient enrollment, allocation, and follow-up is shown in Figure 2. Only one patient in Group A presented with nausea (Figure 2), but this resolved spontaneously within 30 min.

### Demographic variables

No significant differences were found between Groups A and B in patient sex, age, or the laterality of the pc-BPPV (Table 1).

**Table 1:** Patient characteristics.

**Variable**		**Randomized group**		**P-value**
		**Group A**	**Group B**	
Sex	Male	24	26	0.3696
	Female	66	64	
Affected side	Left	42	36	0.1834
	Right	48	54	
Age (years)	Mean	70.7	71.1	0.3959
	Median	72	72	
	Minimum	34	46	
	Maximum	90	88	

### Primary outcome

**Figure 3:**
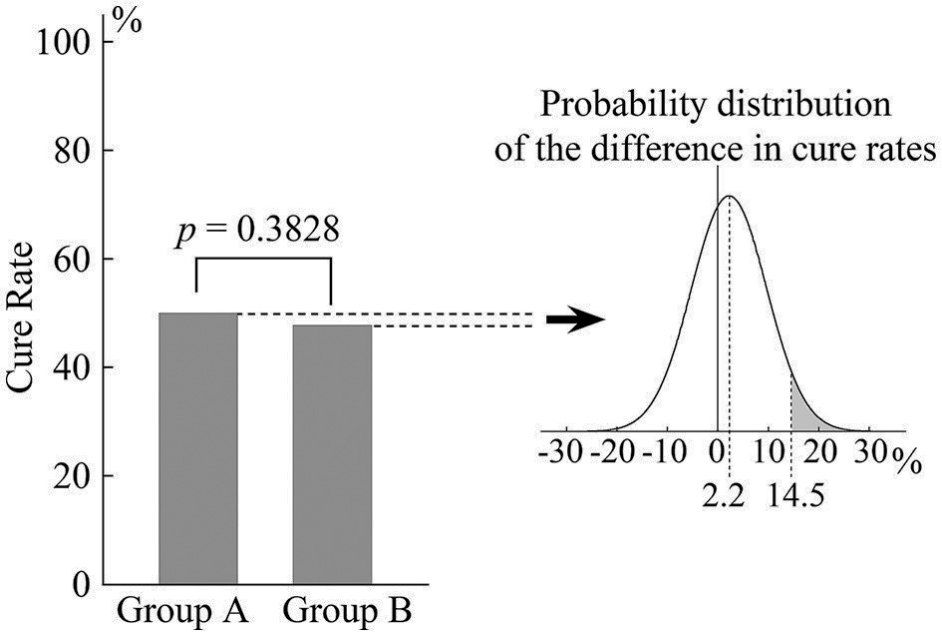
Cure rate for positional nystagmus in patients with pc-BPPV 1 week after treatment. The bar chart shows that there was no significant difference in cure rate between Groups A and B. The probability distribution of the difference is shown in the graph on the right. The gray area represents 5% of the total area and indicates that the upper limit of the 95% confidence interval is 14.5%.

The cure rate was 50.0% (45/90) in Group A and 47.8% (43/90) in Group B. The difference between the groups was not statistically significant (*p* = 0.3828; Figure 3). The graph on the right of Figure 3 shows the probability distribution of the difference in cure rate between the two groups. The gray area represents 5% of the total area of the graph and shows the upper limit of the 95% confidence interval at 14.5%. The non-inferiority margin had been set at 15% *a priori*. These results, therefore, demonstrate the non-inferiority of the repeated DH test to the EM for eliminating positional nystagmus in pc-BPPV after 1 week.

### Secondary outcomes

The secondary outcome results are shown in Table 2. There were no statistically significant group differences in the number of treatment repetitions or distribution of BPPV subtypes after 1 week.

**Table 2:** Secondary outcomes.

**Secondary outcome**		**Randomized group**		**P-value**
		**Group A**	**Group B**	
Number of treatment repetitions	1	72	80	0.1305
	2	16	10	
	3	2	0	
BPPV subtype after 1 week	ipsi pc	45	47	0.5405
	cont pc	1	1	
	lc (geo)	11	5	
	lc (apo)	1	1	

The secondary outcome frequencies are shown for each group.

ipsi pc, BPPV of the posterior canal affecting the ipsilateral side; cont pc, BPPV of the posterior canal affecting the contralateral side; lc (geo), BPPV of the lateral canal with geotropic positional nystagmus; lc (apo), BPPV of the lateral canal with apogeotropic positional nystagmus.

### Classification of evidence

This study was designed to test the hypothesis of non-inferiority of the repeated DH test to the EM for treating patients with pc-BPPV as indicated by the presence or absence of positional nystagmus after 1 week. The study provides class II evidence for the non-inferiority of the repeated DH test; it does not provide class I evidence because the same physician enrolled the patients and performed the follow-up assessments and thus may have remembered the group allocations (although this appears unlikely).

## Discussion

### Primary outcome

This study aimed to show the non-inferiority of the repeated DH test to the EM for treating patients with pc-BPPV. To investigate this, we determined the cure rates for groups of patients who were treated using the repeated DH test or the EM, where “cure” was defined as the absence of positional nystagmus after 1 week. Although a cure for pc-BPPV would involve eliminating otoconial debris from the posterior semicircular canal, this cannot be assessed directly. Therefore, the absence of positional nystagmus was interpreted as showing that the otoconial debris in the posterior semicircular canal had been eliminated (2) and the pc-BPPV was cured. According to the EM theory, pc-BPPV can be cured immediately following the maneuver (7). In this study, we assessed the therapeutic effects of the treatments by examining the positional nystagmus after 1 week; it was considered extremely unlikely that a therapeutic effect would only become apparent after a longer delay. In line with this, previous studies have shown that the EM is effective in eliminating positional nystagmus both up to and after 1 week (6, 21).

In our previous study, we showed that the cure rate for patients with pc-BPPV was 30% higher following the EM than when there was no CRP treatment (19). Based on this, we reasoned that the repeated DH test could be regarded as therapeutic if the cure rate difference with the EM is less than half of 30%, i.e., 15%. We, therefore, set the inferiority margin to be 15% a priori, as recorded in the study protocol (11). As shown in Figure 2, our results revealed that the cure rate difference between the EM and repeated DH tests was 2.2%, with a 95% confidence interval upper limit of 14.5%, i.e., <15%. This, therefore, supports the non-inferiority of the repeated DH test to the EM for treating pc-BPPV.

### Secondary outcomes

The head movements in the EM were designed to move otoconial debris from the posterior semicircular canal to the vestibule. We could, therefore, reason that the number of repetitions needed to eliminate the positional nystagmus should be smaller than for the repeated DH test, where the head movements were not designed to move the otoconial debris in this way. As shown in Table 2, there was no significant difference in the number of treatment repetitions for Groups A and B. This indicates that the lumps of otoconial debris readily disintegrate into smaller parts without the need for many repetitions of the DH test.

We examined the subtypes of BPPV found following the two treatments. Specifically, for the EM, otoconial debris in the posterior semicircular canal could conceivably move to another semicircular canal instead of the vestibule in what is known as a canal switch (18). In this case, another subtype of BPPV would be seen, such as anterior canal BPPV, lc-BPPV with geotropic nystagmus, or lc-BPPV with apogeotropic nystagmus; this would constitute evidence that the otoconial debris moved during the EM. By contrast, a canal switch should not occur during the DH test as the head movements should not move the otoconial debris from between the ampulla of the posterior semicircular canal and the common crus. Therefore, other BPPV subtypes would not be expected to occur following treatment with the DH test. In other words, a canal switch would be expected to occur for Group A (EM) but not Group B (repeated DH test). However, as shown in Table 2, our results did not support this, and there was no significant group difference in the distribution of BPPV subtypes after 1 week. This indicates that the canal switch occurred in both Group A and Group B. In Group A, otoconial debris moved between semicircular canals in the middle of the Epley maneuver, whereas in Group B, the small pieces of otoconial debris possibly moved between canals through the activities of daily living after the repeated DH test but not in the middle of the repeated DH test because the smaller pieces of otoconial debris are more easily moved. Therefore, in Group B patients, whose positional nystagmus disappeared after 1 week, small pieces of otoconial debris might have been returned to the vestibule through the activities of daily living a few days after the repeated DH test.

### Interpretation

Our results demonstrated the non-inferiority of the repeated DH test to the EM for eliminating positional nystagmus in pc-BPPV after 1 week. In a previous study, we showed that the repeated DH test is also non-inferior to the EM for weakening the positional nystagmus after 30 min (22). Therefore, the repeated DH test can be considered to be as effective as the EM for reducing the positional nystagmus after both 30 min and 1 week. The EM was performed to move otoconial debris from the posterior semicircular canal to the vestibule (2). The DH test was repeated to break down the lumps of otoconial debris into smaller parts (this is only the putative pathophysiology) (9, 10). Our results suggest that the disintegration of otoconial debris *via* repeated DH tests has the same therapeutic effect as the transfer of otoconial debris from the posterior canal to the vestibule induced by the EM. The small pieces of otoconial debris may dissolve in endolymph easier than large pieces (23) or potentially return to the vestibule through the activities of daily living, which could account for the equivalent therapeutic effects of the two treatments.

### Generalizability

The patients in this multicenter trial were recruited from various types of hospitals, including municipal hospitals, university hospitals, and critical care medical centers. The patient population in this study is, therefore, likely to be relatively representative of the general population. Of note, patients were not recruited from primary medical care institutions. However, patients with pc-BPPV who present to these centers and remain uncured are generally referred to hospitals, such as those included in our study. Therefore, our study is likely to have included patients who first went to a primary medical care institution. However, if patients had been recruited at primary medical care institutions, the cure rate would likely have been higher because those who are cured within 1 week would have been included in the study. Nevertheless, this should not have affected our main finding that the treatment of pc-BPPV with the repeated DH test is non-inferior to the EM.

### Limitations

To confirm the therapeutic efficacy of the repeated DH test, a better therapeutic effect should be obtained using the repeated DH test compared with a control group of patients with pc-BPPV who do not perform the DH test. However, it is impossible to create such a control group because all patients with pc-BPPV perform the DH test at the time of diagnosis.

A cure rate of 50% was found for Group A in this study. This is lower than that found in other studies that used the EM (24) or Semont maneuver (25), although it is higher than the rate found with no CRP treatment (26). As most of the physicians involved in this study were certified specialists for examining and treating patients with dizziness/vertigo (certified by the Japan Society for Equilibrium Research), this result cannot be attributed to the suboptimal performance of the EM. Instead, the result may relate to the inclusion of patients with pc-BPPV who had been referred from other hospitals and who had already been treated using the EM and yet remained uncured. These patients may be resistant to the EM treatment and thus they may have lowered the cure rate. It is possible that this study included many such patients who are resistant to the EM treatment. However, another possibility for the low cure rate relates to the evaluation criteria used for assessing the elimination of positional nystagmus, which may have been more stringent than in other studies. In this study, we determined that positional nystagmus was present even when it was observed to be very small, and the patient no longer complained of dizziness/vertigo. If this is, indeed, the reason for the low cure rate, this cannot be considered a limitation. After 1 week, the rate of improvement of dizziness/vertigo was 83.3% (75/90) in Group A and 80.0% (72/90) in Group B, which is almost the same as the rates of effectiveness of the EM reported in previous studies (27, 28). Furthermore, the rate of elimination of positional nystagmus on the same day as the EM or the repeated DH test was almost 100% (97.8% (88/90) in Group A and 100% (90/90) in Group B) (Table 2).

## Conclusion

This study demonstrated the non-inferiority of the repeated DH test to the EM for eliminating positional nystagmus in pc-BPPV after 1 week. The elimination of positional nystagmus was considered curative. We propose a putative pathophysiology of the therapeutic effect of the repeated DH test. The disintegration of the lumps of otoconial debris into smaller parts by the repeated DH test has the same therapeutic effect as the transfer of otoconial debris from the posterior canal to the vestibule induced by the EM. Upon disintegration, the smaller pieces of otoconial debris possibly are more easily dissolved in the endolymph and potentially eliminated from the posterior canal, or they might be more easily moved and returned to the vestibule through the activities of daily living.

## Data availability statement

The raw data supporting the conclusions of this article will be made available by the authors, without undue reservation.

## Ethics statement

The studies involving human participants were reviewed and approved by the Ethics Committee of Osaka University Hospital (No. 14128). The patients/participants provided their written informed consent to participate in this study.

## Author contributions

TIm contributed substantially to the conception of the study and drafting of the article. TIm, AU, AY, YT, GS, and KM contributed substantially to the data acquisition. TIm and YU contributed substantially to the data analysis. TIm, SN, KK, TK, KS-H, SM, NK, TN, and AT contributed substantially to the data interpretation. TIg, YO, and TS were substantially involved in the data management center. TIm and AU contributed substantially to the responses to the reviewers' comments. HI and NT contributed substantially to overseeing the study. All authors contributed to the article and approved the submitted version.
